# Syndemics of intimate partner violence among women in HIV endemic South Africa: geospatial analysis of nationally representative data

**DOI:** 10.1038/s41598-022-20230-7

**Published:** 2022-10-27

**Authors:** Andrew Tomita, Diego F. Cuadros, Andrew Gibbs

**Affiliations:** 1 Centre for Rural Health, School of Nursing and Public Health, University of KwaZulu-Natal, Private Bag X7, Durban, South Africa; 2 KwaZulu-Natal Research Innovation and Sequencing Platform (KRISP), College of Health Sciences, University of KwaZulu-Natal, Durban, South Africa; 3 Department of Geography and Geographic Information Science, University of Cincinnati, Cincinnati, OH USA; 4 Digital Epidemiology Laboratory, Digital Futures, University of Cincinnati, Cincinnati, OH USA; 5 Department of Psychology, University of Exeter, Exeter, UK; 6 South African Medical Research Council, Durban, South Africa

**Keywords:** Risk factors, Comorbidities

## Abstract

Despite some improvement in lowering HIV incidence, HIV-related challenges, such as intimate partner violence (IPV), remain unacceptably high among women in South Africa. For decades, researchers and activists have pointed to the complex and intertwined reality of the substance abuse, violence and AIDS (SAVA) syndemic that endangers women. However, more recent systematic review/meta-analysis evidence points to inconclusive association between IPV and alcohol use. Furthermore, much of the evidence is often non-population-based that focuses on the co-occurrence rather than synergistic SAVA interaction. In this study, using the latest data from the South Africa Demographic and Health Survey (SA-DHS), we identified geographic synergistic clustering of IPV associated with HIV and substance abuse in South Africa as a measure of population-level interactions among these factors. The SA-DHS is a nationally representative sample that includes wide-ranging data on health, social challenges and household geo-locations of 5,874 women who participated in the domestic violence module. First, geographical IPV, harmful alcohol use (as the substance abuse measure available in SA-DHS) and HIV clusters were identified using the Kulldorff spatial scan statistic in SaTScan. Second, synergistic interactions related to recent IPV (i.e. recent physical, sexual, emotional violence during the last 12 months) with harmful alcohol use and HIV challenge were measured using RERI [Relative excess risk due to interaction], AP [attributable proportion] and S [Synergy index]. In our results, we spatially identified geographical physical IPV syndemic interactions in parts of the Eastern Cape/Free State Provinces (RERI = 4.42 [95% CI: 2.34–6.51], AP = 0.56 [95% CI: 0.44–0.68], S = 2.77 [95% CI: 2.01–3.84], but not in other forms of IPV. Although IPV, based on decade old concept of SAVA syndemic, was less common/widespread than expected from the national scale population-based data, we identified population-level physical violence syndemic occurring in South Africa. Our study highlights the need to prioritize public health response targeting vulnerable populations residing in these high-risk areas of syndemic mechanisms linking these synergistic epidemics that women face in South Africa.

## Introduction

The global antiretroviral therapy (ART) community scale-up witnessed over the past decade has been a ‘game changer’^1^ that finally turned the tide in the fight against HIV, the disease having claimed countless lives in sub-Saharan Africa (SSA)^2–4^. South Africa, home to one of the largest HIV epidemics in the world (estimated 4.7 million women aged 15 + were living with HIV in 2019^5^), is no exception. According to the latest UNAIDS report in 2020, HIV-related death among women aged 15 + stood at 33,000 cases in 2019^5^, a remarkable achievement, though still unacceptably high, compared to the 120,000 cases in 2005^6^, when the national ART programs (which officially began in late 2003/2004^7^) roll-out was gaining momentum in South Africa.

Despite the population health benefit of ART^8^, HIV incidence remains unacceptably and chronically high among women in South Africa^4^. One of the reasons behind this trend could be the lack of progress in addressing excessively high levels of gender-based violence (GBV). When intimate partner violence (IPV), the most common form of GBV that includes physical, sexual and emotional abuse, is viewed as one barometer of gender inequality, the magnitude of the challenge in South Africa is alarming. According to the 2018 World Health Organization estimate, as many as 24% of women in South Africa experience IPV in their lifetime^9^.

The dual burden of the HIV and IPV epidemics facing women is sadly nothing new. Since the beginning of the global HIV epidemic, public health experts have been warning about the dangers of the substance abuse, violence and HIV/AIDS (SAVA) syndemic, the inseparability of the HIV challenge that is concurring, intersecting and mutually reinforcing the entrenched IPV and substance abuse epidemic^10,11^. Syndemic theory^12^ posits that vulnerable populations often suffer from the effect of synergistic clustering of 2 + population health conditions, which are fueled by macrolevel social, environmental and economic adversities. In the era of the ageing HIV epidemic where the attention to focuses on chronic health challenges (i.e. non-communicable diseases) associated with longer life expectancy with the advent of ART for individuals living with HIV^13^, the challenge of addressing the SAVA syndemic needs to be re-assessed. In light of recent evidence from a systematic review and meta-analysis, which points to no association between IPV and alcohol use (at individual-level)^14^, this calls into question the relevance of decades old concept in SAVA syndemics, with a clear need to reassess whether synergistic clustering of IPV associated with HIV and substance use still matters in South Africa. Furthermore, to the best of our knowledge and literature review, there is no national-level epidemiological evidence that identifies and empirically quantifies the synergistic interactions of IPV associated with HIV and substance use in South Africa at population level to date. As a developing nation in sub-Saharan Africa, South African health system is overburdened and under-resourced^15^, which ultimately requires allocation of limited resources to be targeted to prioritized intervention in vulnerable populations residing in high-risk areas^16^. Towards informing a geographic targeted intervention, we conducted this investigation to first identify geographical IPV syndemic clustering, and then empirically investigate whether HIV and substance use synergistically interact to fuel population-level IPV among women in South Africa.

## Methods

### Data source

We used publicly available cross-sectional data from the South Africa Demographic and Health Survey (SA-DHS)^17^, a nationally representative sample of households that provides unique national-representative insights into population-level socio-demographic profiles and health needs, as well as data on spatial information of the sampled household locations (global position system [GPS] coordinates) which became publicly available in 2019. The SA-DHS employed a stratified, two-stage cluster sampling design to reach a nationally representative sample of households^17^. Briefly, the SA-DHS followed a sampling design with a probability proportional to the sample size of primary sampling units (PSUs) at the first stage, and systematic sampling of residential dwelling units (DUs) in each PSU at the second stage. In terms of data collection, field workers interviewed all households at the selected DUs, with women ages 18 + ever partnered being eligible for the Domestic Violence Module. With an overall response rate of 76%, data on 5,874 (weighted) ever-partnered women were available from the domestic violence module. Thereafter, socio-demographic, clinical and household data were linked to the 5,874 women, which were obtained using other questionnaires [described in the Measures section below]. The use of the data was approved by the University of KwaZulu-Natal Biomedical Research Ethics Committee (00007107).

## Measures

### Health challenges

Three health challenges relevant for the concept of SAVA syndemic, and available from the SA-DHS, were included in our study, namely (1) IPV, (2) substance use, (3) HIV.

1. *Intimate Partner Violence (Domain #1)*
  Recent IPV was the primary focus of our study, with the SA-DHS collecting information on whether types of IPV women had experienced within the past 12 months, based on self-report. Our work focused on three types of IPV, namely: physical, sexual, and emotional violence.
2. *Substance Abuse (Domain #2)*
  According to the WHO^18^, substance abuse refers to the harmful/hazardous use of psychoactive substances, including alcohol. We utilized a standard measure of potential problems with alcohol abuse based on a 4-item questionnaire from the Concern/Cut-down, Anger, Guilt, and Eye-Opener (CAGE) test. Two ‘Yes’ responses in the CAGE were considered to be risk positive for harmful alcohol use^19^.
3. *HIV (Domain #3)*
  The SA-DHS also collected blood samples, with HIV status being determined based on a parallel ELISA (enzyme-linked immunosorbent assay) testing algorithm. The GenScreen HIV 1/2 Combi Assay (Bio-Rad), E411 Cobas HIV 1/2 Combi Assay (Roche), and Geenius HIV1/2 Assay (Bio-Rad) were used for appropriate concordant and discordant confirmatory rapid test results. Further details of the HIV testing algorithm are described in the SA-DHS report^17^.

### Geographical hotspots identification

Geographical clusters for each of the above mentioned three health challenges were identified separately using the Kulldorff spatial scan statistic^20,21^ implemented in SaTScan software^22^. The space permutation model identifies geographical clusters by imposing a circular window with varying radii continuously for each GPS coordinate of the SA-DHS households, and testing whether or not the given health challenge studied is significantly adjacent and unlikely to have arisen by chance, based on a likelihood ratio test. This step of identifying clusters was repeated for each of the abovementioned health challenges and social driver (i.e. poverty). When a geographical cluster was identified (*p* < 0.05), the strength of the cluster (comparing the risks of events inside/outside) was estimated using relative risk (RR). The spatial locations of the geographical cluster identified were mapped using QGIS 3.10.8^23^. Study participants were subsequently classified as exposed to a cluster (i.e. residing in a household located within a cluster) or unexposed (i.e. residing in a household located outside the cluster) as a dichotomized measure.

### Statistical analysis

Three analyses were conducted in our study. First, a descriptive analysis of the participant’s socio-demographics in the Domestic Violence Module was conducted. As the norm of large population-based research, percentages were reported for categorical data. Second, in the event that geographical clusters overlapping IPV, harmful alcohol use and HIV were identified, its additive interaction, which fits the logistic regression (controlling for socio-demographic variables noted in Table 1) were evaluated. This was evaluated based on three measures, namely: RERI [Relative excess risk due to interaction], AP [attributable proportion] and S [Synergy index] and their 95% confidence intervals using the delta method^24,25^. RERI > 0, AP > 0 and S > 1 are indications of interactions, with geographical clusters overlap of IPV, harmful alcohol use and HIV being referred to as ‘IPV syndemic’ in this investigation. Third, we undertook bivariate and multivariable logistic regression to identify significant Socio-demographic correlates of population-level IPV syndemics (residing in areas where all three health challenges overlap). The analyses were adjusted by Domestic Violence Module survey weight provided by SA-DHS, with STATA 17 being used for analysis.

**Table 1:** Sociodemographic of study participants (N = 5,874).

Variable	Categories	Overall	
		n	%
Age	18–24	1041	17.7
	25–34	1573	26.8
	35–44	1117	19
	45–54	773	13.2
	55–64	669	11.4
	65 +	701	11.9
Population group	Black African	4945	84.2
	White	324	5.5
	Coloured	504	8.6
	Indian/Asian and Others	101	1.7
Marital status	Never Union	2243	38.2
	Married	1899	32.3
	Living together	693	11.8
	Widowed	701	11.9
	Divorced and Separated	338	5.8
Educational attainment	No education	497	8.5
	Primary incomplete	614	10.4
	Primary complete	257	4.4
	Secondary incomplete	2289	39
	Secondary complete	1464	24.9
	More than secondary	753	12.8
Employment (past 12 months)	Not employed	4008	68.2
	Employed for cash	1805	30.7
	Employed not for cash	61	1.0
Wealth quintile	Lowest	1096	18.7
	Second	1203	20.5
	Middle	1181	20.1
	Fourth	1164	19.8
	Highest	1230	20.9
Residence type	Urban	3839	65.3
	Rural	2035	34.7

Although the sample size of the Domestic Violence Module indicated in the previous SA-DHS report produced by the South African National Department of Health is 5,865, we report our estimate based on 5874 (weighted) ever-partnered women from the Domestic Violence Module. The main DHS acknowledges the nine cases differential in the SA-DHS report, with the difference after our own reproduction being small.

## Results

### Socio-demographic characteristics

The socio-demographic characteristics of the 5,874 sampled adult females aged 18 + who participated in the domestic violence module are presented in Table 1. With a median age of 40 (IQR = 29–55), most participants were Black African (84.2%), never married (38.2%), unemployed (68.2%) and had not completed secondary school (62.3%). The prevalence of recent experiencing physical, sexual, and emotional violence was 7.8%, 2.3%, and 9.1% respectively.

### SAVA spatial distribution

**Figure 1:**
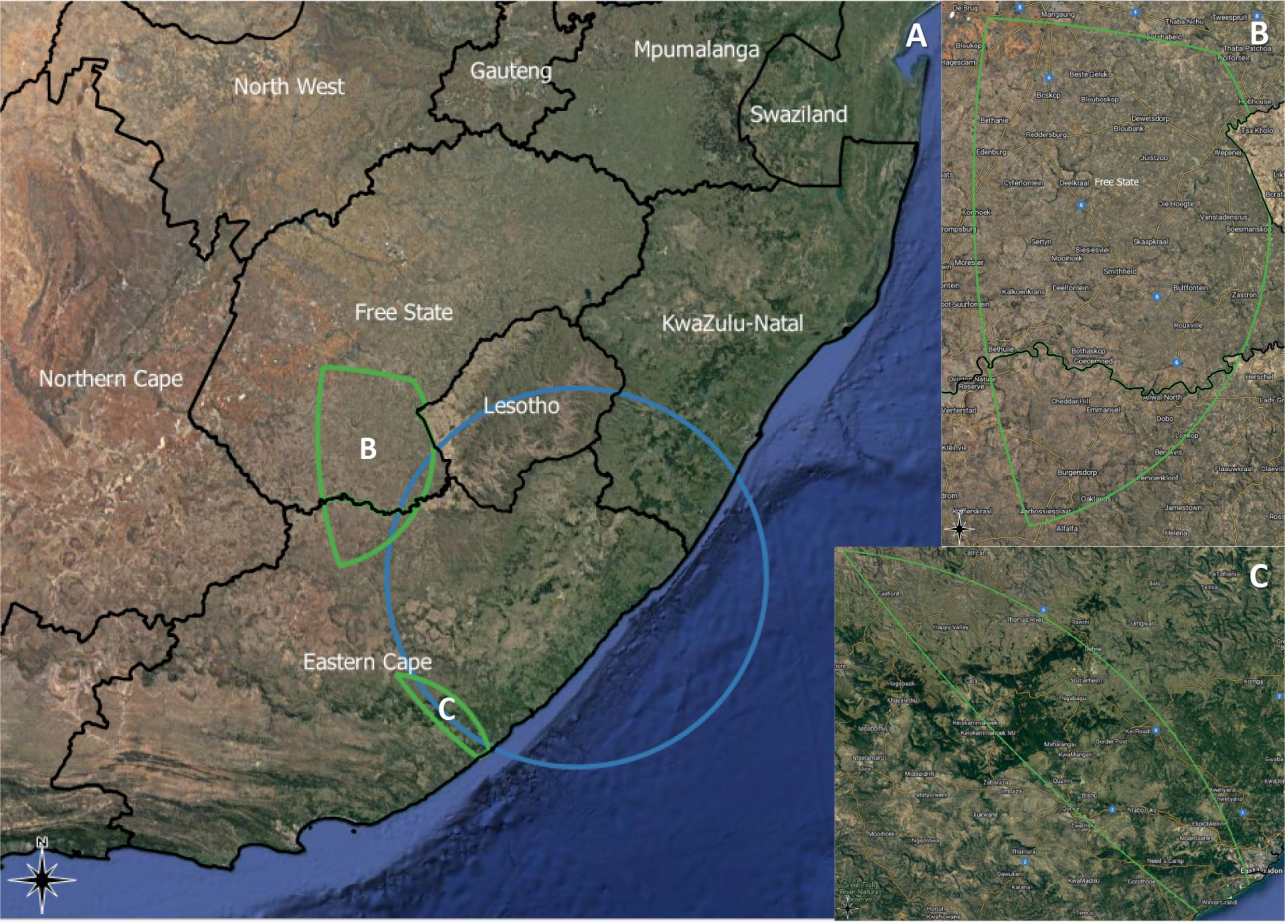
IPV Syndemics in South Africa. Green area indicates intersect of clusters in physical IPV, HIV, and harmful alcohol use where RERI > 0, AP > 0 and S > 1. Light blue indicates cluster of lowest wealth index quintile as a side note in image A. As noted previously, syndemic theory^12^ synergistic clustering of population health conditions has socioeconomic adversities. Area B in image A is magnified in the image B. Area C in image A is magnified in the image C. The map was created using QGIS 3.10 (https://www.qgis.org/en/site/forusers/visualchangelog310/index.html).

The spatial distribution of intersecting geographical clusters in IPV, harmful alcohol use, HIV are illustrated in Fig. 1. We spatially detected (and mapped) geographical physical IPV syndemic interactions (areas where clusters of all three health challenges overlap) in parts of the Eastern Cape/Free State Provinces (RERI = 4.42 [95% CI: 2.34–6.51], AP = 0.56 [95% CI: 0.44–0.68], S = 2.77 [95% CI: 2.01–3.84]. No geographical sexual, or emotional IPV syndemic interactions with significant RERI, AP and S were detected.

### Socio-demographic correlates of IPV syndemics

The socio-demographic profiles of the female study participants residing in physical IPV syndemic area are provided in Table 2. We found that syndemic area is urban and consisted of mostly of Black Africans (98.4%), single marital status (48.6%), incomplete secondary schooling (74.0%) and a middle/high middle wealth index (70.7%). To highlight some of the results of the adjusted regression analyses (Table 3), we found that individuals aged 18–24 [compared to oldest group] (aOR = 5.84, 95% CI: 2.74–12.46), living together [compared to never union] (aOR = 1.53, 95% CI: 1.02–1.47), secondary school incomplete [compared to more than secondary education] (aOR = 2.63, 95% CI: 1.26–5.48), lowest wealth quintile [compared to middle wealth quintile group] (aOR = 1.92, 95% CI: 1.26–2.94) and from urban areas [compared to rural area] (aOR = 1.49, 95% CI: 1.07–2.06) have greater likelihood of living in the identified syndemic area.

**Table 2:** Sociodemographic characteristics and correlates of living inside/outside physical IPV syndemic area based on bivariate analyses (N = 5932).

Variable	Categories	Outside	Inside
		Col	Col
Age	18–24	0.18	0.20
	25–34	0.27	0.20
	35–44	0.19	0.15
	45–54	0.13	0.20
	55–64	0.11	0.16
	65 +	0.12	0.08
Population group	Black African	0.84	0.98
	Non-Black African	0.16	0.02
Marital status	Never Union	0.38	0.49
	Married	0.32	0.23
	Living together	0.12	0.09
	Widowed/Divorced/Separated	0.18	0.20
Educational attainment	Secondary incomplete	0.62	0.74
	Secondary complete	0.25	0.24
	More than secondary	0.13	0.02
Employment (past 12 months)	Not employed	0.68	0.68
	Employed	0.32	0.32
Wealth quintile	Lowest	0.19	0.10
	Second	0.21	0.14
	Middle	0.20	0.27
	Fourth	0.19	0.43
	Highest	0.21	0.05
Residence type	Urban	0.65	1.00
	Rural	0.35	0.00

Relative frequency within column that adds up to 1 (or 100%). Bolded means *p* < 0.05 based on the Pearson chi-squared statistic is corrected for the survey design with the second-order correction of Rao and Scott^44^ and is converted into an F statistic.

**Table 3:** Sociodemographic correlates of living inside/outside physical IPV syndemic area based on logistic regression.

Variable	Categories	aOR	95% CI	
Age: [65 +]	18–24	5.84	2.74	12.46
	25–34	5.81	2.79	12.11
	35–44	5.44	2.63	11.24
	45–54	3.89	1.83	8.24
	55–64	3.90	1.79	8.50
Population group: [Non-Black African]	Black African	0.83	0.53	1.30
Marital Status: [Never Union]	Married	0.80	0.55	1.17
	Living together	1.53	1.02	2.29
	Widowed/Divorced/Separated	0.88	0.53	1.47
Educational Attainment: [More than secondary]	Secondary incomplete	2.63	1.26	5.48
	Secondary complete	2.33	1.12	4.86
Employment (past 12 months): [Not employed]	Employed	0.97	0.71	1.32
Wealth Quintile: [Highest]	Lowest	1.92	1.26	2.94
	Second	1.03	0.71	1.49
	Middle	0.83	0.56	1.24
	Fourth	0.51	0.28	0.92
Residence Type: [Rural]	Urban	1.49	1.07	2.06

## Discussion

Based on a nationally representative community sample in South Africa, our study empirically identified geographic synergistic clustering of physical IPV associated with HIV and alcohol use (although not with emotional or sexual IPV), suggesting a population-level interaction among these factors. Although more recent systematic review/meta-analysis evidence point to an inconclusive association between IPV and alcohol use^14^, our investigation driven by nationally-representative population-based approach, makes an important contribution that speaks to the relevancy of the decade-old concept of SAVA syndemics since the onset of the HIV epidemic^26,27^. Although some progress are made in lowering of HIV incidence in South Africa^8^, unacceptably high level of IPV that exist in South Africa requires urgent public health action to addresses “intertwined and mutual enhancing epidemics”^28^ of substance abuse, and violence (in addition to HIV), particularly in areas where all these health challenges collide identified in this study.

A syndemic that methodologically requires a vast number of population-based samples, along with detailed geospatial data to construct different disease clusters, remains an under-investigated topic in resource-limited settings. To the best of our knowledge, this is the first study in SSA that attempted to identify the spatial variability of syndemic health challenges, including the population-based IPV syndemic, based on the concept of SAVA syndemics, at a national scale, thus making comparison difficult with other studies. Nonetheless, a recent large population-based South African multi-morbidity study in a high HIV prevalence rural setting (at the time of this report) identified similar findings of the convergence of an infectious and non-communicable disease epidemic setting, although no measures of substance use, including harmful alcohol use, were available^29^.

IPV spatial synergistic clustering in parts of the Eastern Cape and Free State Provinces warrants further discussion. In Eastern Cape, these were detected in SA-DHS households from the western part of East London (i.e. Wilsonia) towards Qonce/Zwelitsha, passing through Mdantsane via the N2 National Road. According to the Buffalo City Municipal Spatial Development Framework report of 2020^30^, there are a number of designated informal settlements located in Wilsonia, Mdantsane, and Zwelitsha along and adjacent to the N2 National Road corridor. In the Free State, IPV spatial synergistic clusters were detected in SA-DHS households from the urban nodes/centers of Dewetsdorp to the southern parts of Manguang via the Regional Route R702. According to the Mangaung Municipal Spatial Development Framework report of 2020^31^ there are number of highlighted informal settlements in Mangaung and Dewetsdorp close to Regional Route R702. Although we cannot fully pinpoint that spatial synergistic clusters of IPV given that the DHS randomly displaces the urban GPS positions for a maximum of two kilometers to protect participant confidentiality^32^ (DHS, 2013), the unique and unfortunate legacy of informal settlements cannot easily be dismissed^33^. Informal settlements, high population density areas at the outskirts of urban areas and other economic centers in South Africa often lack formal built infrastructure, and have little or no access to basic services, such as water, electricity and sanitation^34^. These areas exist historically (and continue to exist) as consequences of apartheid regime’s urban planning policy, which neglected the housing needs of migrants that industries relied upon for the country’s economic development^35^.

Although it could easily be argued that the detection of spatial synergistic cluster near the national roadway, or adjacent to the province’s economic hubs, can be attributed to ease of sampling by SA-DHS, other geospatial studies from South Africa also note similar challenges in informal settlements that are historically, and continues to be neglected. For example, several South African studies from the Africa Health Research Institute Demographic and Health Surveillance System (2016–2019), located in the Umkhanyakude District of the northern KwaZulu-Natal Province, point to clusters of HIV infections in informal settlement communities near the National Road that links the city of Durban with Mozambique, Swaziland and Mpumalanga Province in the north^36,37^. In this surveillance area, HIV incidence among young women is persistently high (5.12, 95% CI: 4.25–6.37)^38^. In the current investigation, we found that study participants residing in IPV spatial synergistic cluster communities were mainly young, Black African females (98.4%) often single (the largest group being ages 18–24: 20.4%), unemployed (68.1%), and had not completed secondary school (74.0%). Our study highlights the need to prioritize public health responses for socioeconomically vulnerable young women in the high-risk syndemic areas in South Africa.

### Limitations

There are several limitations that warrant discussion. First, not all health challenges of the study participants were based on laboratory or diagnostic tests. Given the vast number of study participants and health domains available for assessment in the SA-DHS, it is common in large population-based studies to rely on self-reported data. We acknowledge the possible underreporting of health and IPV challenges in our study, as is commonly detected in other population-based studies^39–42^. Second, our study was based on a cross-sectional design, with further studies being needed to establish temporal relationships between syndemics and IPV. Notwithstanding these limitations, this investigation identified the spatial heterogeneity of health syndemics at a national scale in South Africa, with a significant link between these syndemic health challenges and IPV among women.

## Conclusion

Although IPV, based on decade old concept of SAVA syndemic, was less common/widespread than expected from the nationally representative population-based data, we nonetheless identified physical violence syndemic occurring in South Africa which speaks to the relevance of intertwined reality of the substance abuse, violence and AIDS (SAVA) syndemic in South Africa. In recent years, there has been a growing call for people-centered policies^43^ that address complex challenges individual face in their respective community. The findings from our study, which geographically identified at-risk IPV syndemic communities, are an essential first step towards implementing people-centered interventions in the era of an aging HIV epidemic. Although more further studies are needed about the role of informal settlements, public health intervention should (re-)focus on alcohol reduction and HIV prevention at-risk impoverish communities that drive IPV among women in South Africa.

## References

[1] De Cock KM, El-Sadr WM, Ghebreyesus TA (2011). Game changers: Why did the scale-up of HIV treatment work despite weak health systems?. J. Acquir. Immune Defic. Syndr..

[2] Frank TD, Carter A, Jahagirdar D (2019). Global, regional, and national incidence, prevalence, and mortality of HIV, 1980–2017, and forecasts to 2030, for 195 countries and territories: A systematic analysis for the Global Burden of Diseases, Injuries, and Risk Factors Study 2017. Lancet HIV..

[3] Reniers G, Slaymaker E, Nakiyingi-Miiro J (2014). Mortality trends in the era of antiretroviral therapy: Evidence from the network for analysing longitudinal population based HIV/AIDS data on Africa (ALPHA). AIDS.

[4] Birdthistle I, Tanton C, Tomita A (2019). Recent levels and trends in HIV incidence rates among adolescent girls and young women in ten high-prevalence African countries: A systematic review and meta-analysis. Lancet Glob Heal..

[5] UNAIDS. UNAIDS Data 2020. Accessed August 24, 2021. https://www.unaids.org/sites/default/files/media_asset/2020_aids-data-book_en.pdf..

[6] UNAIDS UNAIDS Data 2018. Accessed August 24, 2021. https://www.unaids.org/en/resources/documents/2018/unaids-data-2018

[7] Johnson LF (2012). Access to antiretroviral treatment in South Africa 2004–2011. South Afr J HIV Med..

[8] Tanser F, Bärnighausen T, Grapsa E, Zaidi J, Newell ML (2013). High coverage of ART associated with decline in risk of HIV acquisition in rural KwaZulu-Natal, South Africa. Science.

[9] World Health Organization. Violence against women prevalence estimates, 2018. Global, regional and national prevalence estimates for intimate partner violence against women and global and regional prevalence estimates for non-partner sexual violence against women. Accessed August 24, 2021. https://cdn.who.int/media/docs/default-source/documents/violence-prevention/vaw_report_web_09032021_oleksandr.pdf.

[10] Singer M (2000). A dose of drugs, a touch of violence, a case of AIDS: Conceptualizing the Sava Syndemic. Free Inquiry Creat. Sociol..

[11] Singer M, Clair S (2003). Syndemics and public health: Reconceptualizing disease in bio-social context. Med Anthropol Q..

[12] Tsai AC, Mendenhall E, Trostle JA, Kawachi I (2017). Co-occurring epidemics, syndemics, and population health. Lancet.

[13] Lancet HIV (2017). Preparing for an ageing HIV epidemic. Lancet HIV..

[14] Bacchus LJ, Ranganathan M, Watts C, Devries K (2018). Recent intimate partner violence against women and health: A systematic review and meta-analysis of cohort studies. BMJ Open.

[15] Health Systems Trust. South African Health Review 2021: Health sector responses to COVID-19. Accessed on August 25, 2022. https://www.hst.org.za/publications/South%20African%20Health%20Reviews/SAHR21_WEB_NoBlank_sm_24022022_OD.pdf.

[16] Tomita A, Vandormael AM, Bärnighausen T, Phillips A, Pillay D, de Oliveira T, Tanser F (2019). Sociobehavioural and community predictors of unsuppressed viral load in a hyper-endemic rural African community: Multilevel results from a population-based viral load survey. AIDS.

[17] National Department of Health, Statistics South Africa, South African Medical Research Council, and ICF. South Africa Demographic and Health Survey 2016. Accessed August 24, 2021. https://dhsprogram.com/pubs/pdf/FR337/FR337.pdf.

[18] World Health Organization. Substance Abuse. Accessed July 13, 2022. https://www.afro.who.int/health-topics/substance-abuse.

[19] Ewing JA (1984). Detecting alcoholism: The CAGE questionnaire. JAMA.

[20] Kulldorff M (1997). A spatial scan statistic. Commun. Stat. Theory Methods..

[21] Kulldorff M, Heffernan R, Hartman J, Assuncao R, Mostashari F (2005). A space-time permutation scan statistic for disease outbreak detection. PLoS Med..

[22] Kulldorff M. SaTScan v9.0: Software for the Spatial and Space-Time Statistics. 2010. National Cancer Institute.

[23] QGIS. QGIS Geographic Information System. 2021. QGIS Association.

[24] Hosmer DW, Lemeshow S (1992). Confidence interval estimation of interaction. Epidemiology.

[25] Andersson T, Alfredsson L, Källberg H, Zdravkovic S, Ahlbom A (2005). Calculating measures of biological interaction. Eur. J. Epidemiol..

[26] Gilbert L, Raj A, Hien D, Stockman J, Terlikbayeva A, Wyatt G (2015). Targeting the SAVA (Substance Abuse, Violence, and AIDS) syndemic among women and girls: A global review of epidemiology and integrated interventions. J. Acquir. Immune Defic. Syndr..

[27] Meyer JP, Springer SA, Altice FL (2011). Substance abuse, violence, and hiv in women: A literature review of the syndemic. J. Women's Health.

[28] Singer M (1994). AIDS and the public health crisis of the U.S. urban poor: The perspective of critical medical anthropology. Soc Sci Med..

[29] Wong EB, Olivier S, Gunda R (2021). Convergence of infectious and non-communicable disease epidemics in rural South Africa: A cross-sectional, population-based multimorbidity study. Lancet Glob Heal..

[30] Buffalo City Metropolitan Municipality. Municipality Spatial Development Framework 2019–2024. Accessed on July 13, 2022. https://afesis.org.za/wp-content/uploads/2020/10/BCMM-SDF-Final-Draft.pdf.

[31] Mangaung Metropolitan Municipality. Spatial Development Framework (SDF) 2020. Accessed on July 13, 2022. http://www.mangaung.co.za/wp-content/uploads/2020/08/Mangaung-Spatial-Development-Framework-2020.pdf.

[32] ICF. GPS Data Collection. Accessed on July 13, 2022. https://dhsprogram.com/methodology/GPS-Data-Collection.cfm.

[33] Oyekunle V, Tomita A, Gibbs A (2022). High levels of poor mental health among young men in urban informal settlements in South Africa: A community-based study of social determinants. Psychol Health Med..

[34] Socio-Economic Rights Institute of South Africa. Informal settlements and human rights in South Africa. Accessed on July 13, 2022. https://www.ohchr.org/sites/default/files/Documents/Issues/Housing/InformalSettlements/SERI.pdf.

[35] Marais L, Cloete J, Denoon-Stevens S (2018). Informal settlements and mine development: Reflections from South Africa's periphery. J. South Afr. Inst. Min. Metall..

[36] Tanser F, Bärnighausen T, Cooke GS, Newell ML (2009). Localized spatial clustering of HIV infections in a widely disseminated rural South African epidemic. Int. J. Epidemiol..

[37] Tanser F, Bärnighausen T, Dobra A, Sartorius B (2018). Identifying 'corridors of HIV transmission' in a severely affected rural South African population: A case for a shift toward targeted prevention strategies. Int. J. Epidemiol..

[38] Akullian A, Vandormael A, Miller JC, Bershteyn A, Wenger E, Cuadros D, Gareta D, Bärnighausen T, Herbst K, Tanser F (2021). Large age shifts in HIV-1 incidence patterns in KwaZulu-Natal, South Africa. Proc. Natl. Acad. Sci. U.S.A..

[39] Mooney AC, Campbell CK, Ratlhagana MJ (2018). Beyond social desirability bias: Investigating inconsistencies in self-reported HIV testing and treatment behaviors among HIV-positive adults in North West Province, South Africa. AIDS Behav..

[40] Fishel JD, Barrère B, Kishor S. Validity of data on self-reported HIV Status in Malawi and Uganda and implications for measurement of ARV Coverage. DHS Methodological Reports No. 10. Accessed August 24, 2021. https://dhsprogram.com/pubs/pdf/MR10/MR10.pdf.

[41] Rohr JK, Gómez-Olivé FX, Rosenberg M (2017). Performance of self-reported HIV status in determining true HIV status among older adults in rural South Africa: A validation study. J. Int. AIDS Soc..

[42] Machisa MT, Christofides N, Jewkes R (2017). Mental ill health in structural pathways to women’s experiences of intimate partner violence. PLoS ONE.

[43] World Health Organization. WHO global strategy on integrated people-centred health services 2016–2026. Accessed on July 13, 2022. https://interprofessional.global/wp-content/uploads/2019/11/WHO-2015-Global-strategy-on-integrated-people-centred-health-services-2016-2026.pdf.

[44] Rao JNK, Scott AJ (1984). On Chi-squared tests for multiway contingency tables with cell proportions estimated from survey data. Ann. Stat..

